# BRCA2 germline mutation in familial leukaemia with familial breast cancer: a case report

**DOI:** 10.1007/s00277-023-05546-2

**Published:** 2023-11-20

**Authors:** Jing Wang, Heyang Zhang, Rui Zhang

**Affiliations:** Department of Hematology, The First Hospital of China Medical University, Shenyang, 110001 China

Dear Editor,

With germline susceptibility to myeloid malignancies included in the updated leukaemia classification scheme for the first time, in 2016, the World Health Organization (WHO) designated familial haematopoietic malignancies as an essential component of leukaemia diagnosis [1]. The currently reported familial aggregation of haematological malignancies is mainly chronic lymphocytic leukaemia (CLL), acute myeloid leukaemia (AML), lymphoma and multiple myeloma (MM). The aggregation is not obvious in Burkitt lymphoma and acute lymphoblastic leukaemia (ALL) [2]. In this study, we present a case of *BRCA2* germline mutation in familial ALL with familial breast cancer, which has not been reported before.

A 58-year-old Chinese woman admitted to our hospital on August 11, 2021, due to elevated white blood cells for 3 days. One and a half months before admission, the patient underwent a surgery with modified radical mastectomy of the right breast and the pathological evaluation of the specimens revealed invasive breast cancer, grade II, with no vascular invasion. A bone marrow biopsy was performed, and the diagnosis was confirmed as early T-cell precursor acute lymphoblastic leukaemia (ETP-ALL). Whole transcriptome sequencing revealed no fusion gene but mutations in *NOTCH-1*, *KRAS*, *EPPK1*, *NF1*, *DNMT3A*,*TTN*, and *BRCA2*. The patient refused endocrine oral drugs for breast carcinoma therapy. She refused allogeneic haematopoietic stem cell transplantation (allo-HSCT) for treatment of ALL, so the CCLG-ALL 08 protocol was given.

Her final disease-free survival (DFS) and overall survival (OS) were 6 months and 16 months, respectively.

Her family history included breast cancer and T-ALL in her younger sister. On February 1, 2009, a mass in the right neck and routine blood tests was detected in the patient’s sister (45 years old). She was positive for the fusion gene *BCR/ABL P210*, and she was finally considered to have Ph + T ALL. The patient was given VDP chemotherapy for 1 course but refused further treatment. Half a year later, the patient had a breast mass, as indicated by pathological examination in another hospital. The pathological findings confirmed breast cancer, and she refused treatment and eventually died of disease progression.

**Fig. 1:**
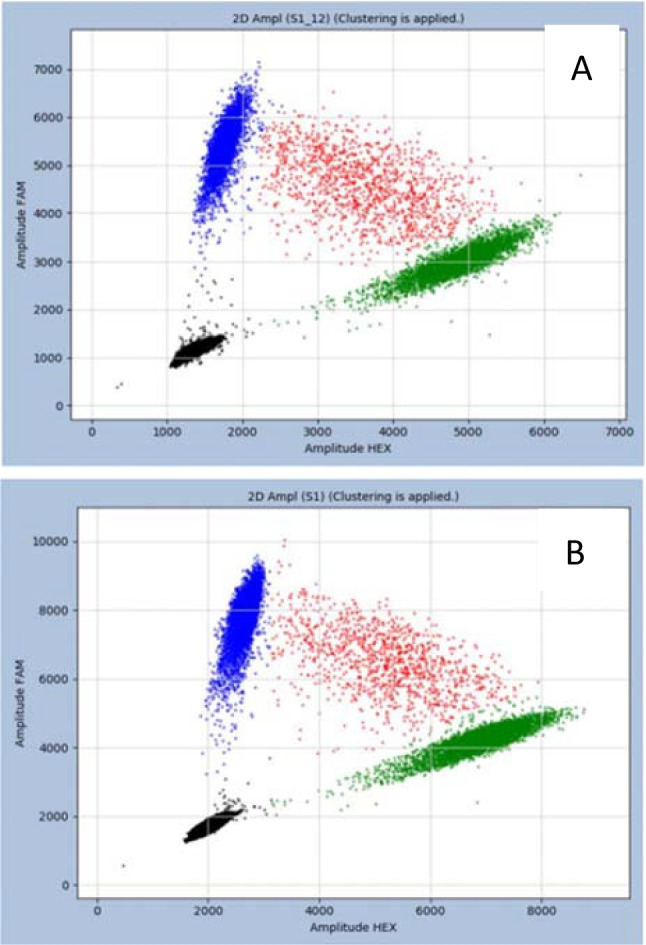
The blue part is pure mutant, the green part is pure wild-type, and the red part is mutant and wild-type double-positive micropores. The positive gene includes the blue and red part. **A** The patient’s oral mucosaDigital-PCR of *BRCA2-P89L* (49.948%). **B** Her son’s oral mucosaDigital-PCR of *BRCA2-P89L* (49.440%)

For verification, the oral mucosa of the patient and her son were examined for mutations in *NOTCH1* and *BRCA2* by digital quantification PCR to determine whether the presence of germline mutations. Both of them were negative for *NOTCH1* mutation and positive for *BRCA2* mutation (Fig. 1A, B). The proportion of *BRCA2-P89L* mutations was 49.948% in the patient and 49.440% in her son, indicating that the *BRCA2* gene mutation was a germline mutation. Combined with the above results and diagnostic criteria, we comprehensively considered the patient to be diagnosed with familial leukaemia with familial breast cancer.

It is well known that breast cancer is inherited. Secondary tumours of breast cancer are also common. The most common haematological disease after breast cancer is treatment-induced MDS/AML [3]. Familial ALL is rare, but there is a cytogenetic basis for the pathogenesis. The same germline gene mutation is a common characteristic of familial tumours, with implications for their close and extended relatives [4]. Some germline mutations have been reported to be associated with familial leukaemia, including *IKZF1*, *PAX5*, *ETV6*, *SH2B3*, and *TYK2*, among others [5]. In the research of Li X [6], the patterns of increased familial cancers suggest that *BRCA2* mutations might contribute to associations of ALL with breast and prostate cancers. Breast cancer is one of the associated cancers in families of very young ALL patients, and the Fanconianaemia-*BRCA2* pathway is a known risk factor for childhood ALL [6]. *ETV6* and *NOTCH1* germline variants may have contributed to the malignant transformation to ALL in the report of Dirse V [7]. Therefore, we hypothesized that this patient had familial breast cancer with familial leukaemia with *BRCA2* mutation.

*BRCA2* germline gene mutations are not only hotspot mutations of familial breast cancer but also the cause of secondary tumours in this case. *BRCA2*-deficient mice spontaneously develop T-lymphoblastic malignancies at high frequency [8]. Roy U et al. considered novel oncogenic mutations, including *TP53*, *BRCA2*, *NOTCH1*, *DNMT3A,* and an array of other genes, to disrupt genetic and epigenetic homeostasis in T-ALL [8]. Therefore, we speculate that driven by *BRCA2* mutations, synergistic gene mutations such as *NOTCH1* and *DNMT3A* promote the occurrence and development of ALL. At the same time, we noticed that the sister was diagnosed with Ph + T-ALL (*BCR/ABL P210* +), which is very rare since the incidence is only 2% in T-ALL. Only 30 cases have been reported, and the clinical characteristics, underlying genetic lesions, treatment strategies, and prognosis are still unclear [9].

For many leukaemia predisposition disorders of MDS or acute leukaemia (AL), the curative therapy of choice is allogeneic HSCT. It is noted that transplant donors who are at risk of familial leukaemia should be avoided. Since familial haematologic malignancies have poor prognosis and are prone to relapse and progression, clinicians should pay attention to detailed investigation of the family history of patients and conduct follow-up monitoring. The role and importance of germline mutations in familial cancer need more research.
